# Engineering an Anti-Transferrin Receptor ScFv for pH-Sensitive Binding Leads to Increased Intracellular Accumulation

**DOI:** 10.1371/journal.pone.0145820

**Published:** 2015-12-29

**Authors:** Benjamin J. Tillotson, Loukas I. Goulatis, Isabelle Parenti, Elizabeth Duxbury, Eric V. Shusta

**Affiliations:** University of Wisconsin-Madison, Dept. of Chemical and Biological Engineering, Madison, WI, United States of America; National Cancer Institute, NIH, UNITED STATES

## Abstract

The equilibrium binding affinity of receptor-ligand or antibody-antigen pairs may be modulated by protonation of histidine side-chains, and such pH-dependent mechanisms play important roles in biological systems, affecting molecular uptake and trafficking. Here, we aimed to manipulate cellular transport of single-chain antibodies (scFvs) against the transferrin receptor (TfR) by engineering pH-dependent antigen binding. An anti-TfR scFv was subjected to histidine saturation mutagenesis of a single CDR. By employing yeast surface display with a pH-dependent screening pressure, scFvs having markedly increased dissociation from TfR at pH 5.5 were identified. The pH-sensitivity generally resulted from a central cluster of histidine residues in CDRH1. When soluble, pH-sensitive, scFv clone M16 was dosed onto live cells, the internalized fraction was 2.6-fold greater than scFvs that lacked pH-sensitive binding and the increase was dependent on endosomal acidification. Differences in the intracellular distribution of M16 were also observed consistent with an intracellular decoupling of the scFv M16-TfR complex. Engineered pH-sensitive TfR binding could prove important for increasing the effectiveness of TfR-targeted antibodies seeking to exploit endocytosis or transcytosis for drug delivery purposes.

## Introduction

Receptor-ligand recognition and binding frequently depend on pH-induced changes stemming from the combined protonation states of amino acids within the protein. Histidine is considered a key amino acid driving pH sensitivity having a side-chain pKa of 5.5–6.5 in the context of proteins [1]. Evidence suggests that proteins have adapted to function in a range of subcellular pH environments through non-random placement of histidine residues [2]. These phenomena have been exploited in therapeutic protein design to alter intracellular trafficking. For example, interactions with the neonatal Fc-receptor (FcRn), which functions in a pH-dependent manner to regulate serum IgG levels [3], have been modified. The Fc region surrounding critical histidine residues of the monoclonal antibody Motavizumab was mutated improving FcRn binding at pH 6.0 without affecting its affinity at pH 7.2, thereby achieving a 4-fold extension in serum half-life [4,5,6]. In contrast, desiring a reduction in therapeutic IgG serum half-life, a competitive antibody, or “Abdeg”, was created to bind FcRn tightly at both pH 6.0 and pH 7.2, hence occupying FcRn at the expense of therapeutic antibody binding [7]. While these studies describe the modulation of a preexisting pH-dependent system, it is also possible to introduce pH-sensitive binding. As examples, both the anti-IL6R antibody Tocilizumab [8], and the anti-PCSK9 antibody RN316 [9] were engineered to escape target-mediated degradation by introducing histidine residues at select positions in the antibody CDR loops, so as to induce antibody-antigen dissociation at endosomal pH. Engineering pH-sensitive ligand binding has also been employed to increase the potency of non-immunoglobulin scaffolds as in the case of the cytokine GCSF [10], and the iron carrier protein transferrin [11].

The transferrin receptor (TfR) presents a valuable therapeutic target which can be antagonized directly, or exploited indirectly as an intracellular drug delivery vector. These opportunities result from the ubiquitous expression of TfR on normal cells and elevated expression on cancer cells, as well as the endocytotic route used to transport iron-bearing transferrin inside the cell (reviewed in [12,13]). The natural ligand for TfR, the serum protein transferrin (Tf), circulates in iron-free (apoTf) or iron-bound (holoTf) forms [14,15]. HoloTf binds the transferrin receptor (TfR) tightly at blood pH (7.2–7.4), and the complex is internalized via clathrin-mediated endocytosis (CME) [16]. As holoTf-TfR complexes cycle though acidic endosomes (pH 5.0–6.0), an intricately coordinated series of pH-induced conformational changes induces the release of both iron molecules to yield apoTf, which has an increased affinity for TfR at endosomal pH [15,17,18,19]. This is followed by recycling of the apoTf-TfR complex to the cell surface (pH 7.2–7.4) where apoTf has a decreased affinity for TfR and dissociates back into the blood stream [17,20]. Cytotoxins based on conjugates of transferrin have been widely studied as therapeutic agents [21]. A detailed kinetic model of the TfR cycle was created and analyzed for routes that might lead to a greater overall cellular association of Tf or Tf conjugates [11]. It was posited that inhibition of iron release from Tf could lead to endosomal dissociation of holoTf that, unlike apoTf, could rapidly rebind at blood pH and participate in further cycles of endocytosis at blood pH [11,17]. Indeed, when Tf was genetically altered to inhibit iron release, diphtheria toxin conjugates of the mutant Tf showed increased cytotoxicity compared to wild-type Tf conjugates [22]. Similarly, it has been shown that improved cytotoxin efficacy for Tf conjugates as well as anti-TfR antibodies is a direct result of increased cellular association [23,24,25].

Here we reasoned that the intracellular accumulation of an anti-TfR antibody could be increased by engineering enhanced dissociation from TfR at endosomal pH, thereby decoupling antibody uptake from post-internalization TfR trafficking dynamics. To test this hypothesis, an anti-TfR single-chain antibody (scFv) was subjected to histidine-saturation mutagenesis at a single CDR known to participate in TfR binding, and the resultant library was screened. These methods resulted in an scFv, M16, that exhibited rapid dissociation at endosomal pH, while maintaining a high affinity for TfR at neutral pH. When dosed onto proliferating cancer cells, M16 displayed greater total cellular association than the wild-type scFv H7, which could be attributed to elevated intracellular accumulation. Immunocytochemical analysis revealed markedly different patterns of accumulation for scFv M16, indicating a departure in trafficking from the canonical Tf-TfR pathway.

## Materials and Methods

### Cells, media, and plasmids

SK-BR-3 cells (HTB-30) were obtained from American Type Culture Collection (ATCC) and maintained at 37°C, 5% CO2, in growth medium (SKBR3GM) composed of McCoy’s 5A basal media (10050CV, Corning-Cellgro) supplemented with 10% fetal bovine serum (Gibco) and 1x antibiotic/antimycotic solution (PSA, Gibco). *Saccharomyces cerevisiae* strains EBY100 [26] and AWY100 [27] were used for surface display, while strain YVH10 [28] was used for soluble scFv secretion. The vector pCT-ESO [29] was the backbone for all scFv surface-display experiments, while plasmid pRS316-GAL-4420 [30] provided the backbone for scFv secretion. EBY100 were grown in SD-CAA (20 g/L dextrose, 6.7 g/L yeast nitrogenous base, 100mM sodium phosphate buffer pH 6.0, 5.0 g/L bacto-casamino acids lacking tryptophan and uracil). AWY100 was grown in SD-CAA supplemented with 40 mg/L uracil. YVH10 were grown in SD-2xSCAA+Trp (20 g/L dextrose, 6.7 g/L yeast nitrogenous base, 100 mM sodium phosphate buffer pH 6.0, 190 mg/L Arg, 108 mg/L Met, 52 mg/L Tyr, 290 mg/L Ile, 440 mg/L Val, 220 mg/L Thr, 130 mg/L Gly, 40 mg/L Trp, lacking leucine and uracil). Induction medium (SG-CAA or SG-2xSCAA) was composed of SD-CAA or SD-2xSCAA with 20 g/L D-galactose instead of dextrose. Solid media for individual colony selection were made identically to the above yeast media recipes with the addition of 15 g/L bacto-agar and 180 g/L d-sorbitol. During FACS, sorted EBY100 were collected into SD-CAA supplemented with 50 mg/L kanamycin sulfate (K4378, Sigma-Aldrich) to prevent bacterial growth.

### Histidine-saturation mutagenesis of scFv H7 CDRH1 and library construction

**Fig 1:**
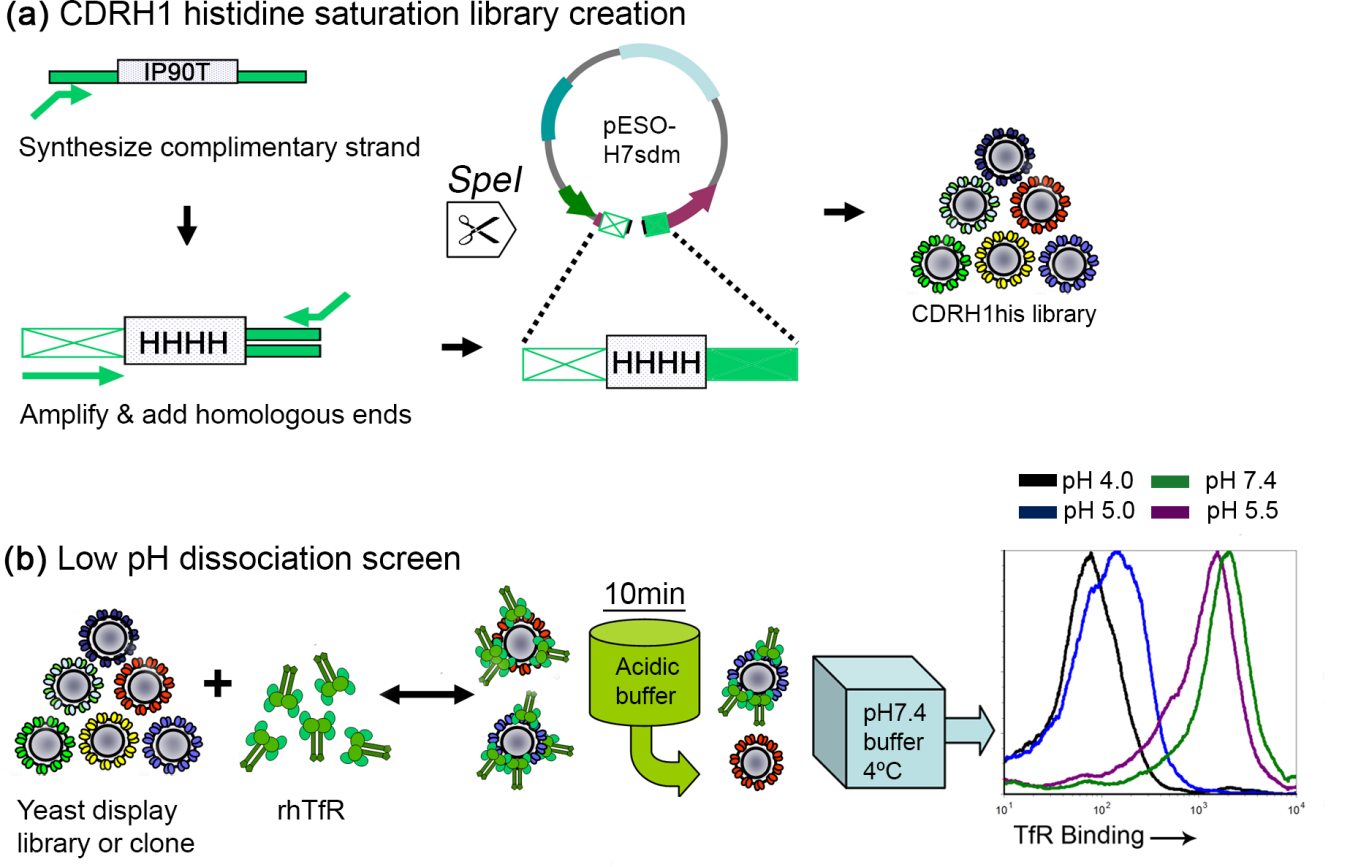
Creation and screening methodology for a histidine-saturated CDRH1 library based on the wild-type anti-TfR scFv, H7. (a) The pESO-H7sdm vector contained a mutant scFv H7, that was specially designed to harbor a unique restriction site (*SpeI*) followed by two stop codons in CDRH1. *SpeI* digestion of pESO-H7sdm produced a linearized backbone that would be undetectable by flow cytometry if introduced as a reclosed vector into yeast (no c-*myc* epitope tag expression as a result of the double stop codon). A double-stranded DNA cassette containing the histidine-saturated CDRH1 was built using two cycles of PCR, from primers and a degenerate oligonucleotide (IP90T). Although depicted as a single entity for simplicity, IP90T was in fact a mixture of millions of unique ssDNA oligos representing all possible combinations of histidine in CDRH1. The CDRH1his cassette and linearized pESO-H7sdm were used to create the CDRH1his library by homologous recombination in yeast. (b) Screening and assessment of yeast-displayed scFvs was accomplished by saturating with recombinant human transferrin receptor (rhTfR) followed by a 10 minute incubation in acidic buffer (pH 4.0–6.5) and assay for TfR dissociation. A pH 5.5 buffer was used to simulate endosomal conditions relevant to transferrin-TfR dissociation [19,20]. A dissociation control was included by using a pH 7.4 buffer as opposed to acidic buffer at this step. The dissociation process was halted by addition of an excess volume of ice cold pH 7.4 buffer. Surface constructs were subsequently immunolabeled and analyzed by flow cytometry. The histogram depicts the response of yeast-displayed wild-type scFv H7 to 10 minute incubation with buffers having acidic pH.

The scFv H7 against human transferrin receptor was a kind gift from Dr. James Marks at the University of California-San Francisco [31]. The negative control for surface display and soluble scFv assays was an anti-hen egg lysozyme antibody, scFv D1.3 [32]. scFv H7 was previously subcloned into the pCT-ESO backbone to create the plasmid pESO-H7 [33]. pESO-H7 formed the basis for constructing a recombinant, histidine-saturation library focused on scFv H7 CDRH1. To allow homologous recombination (and to preemptively silence any yeast receiving reclosed vector during the transformation), site-directed mutagenesis was used to create a unique restriction enzyme site (SpeI) followed by a double stop codon in the middle of CDRH1. Briefly, Phusion DNA polymerase (NEB) was used to linearly amplify and mutate the pESO-H7 template with primers H7sdmF 5’-GCCTCTCGATTCACCTTCACTAGTTAATAAATGCACTGGGTCCGC-3’ and H7sdmR 5’-CTGGCGGACCCAGTGCATTTATTAACTAGTGAAGGTGAATCGAGAGGCCAG-3’. The resulting PCR product was digested with DpnI (NEB), ethanol precipitated in the presence of sodium acetate and pellet paint (Covagen), rehydrated in 1x TE, and digested with DpnI a second time. After successful transformation into XL1 Blue Supercompetent cells (Agilent) following the manufacturer's protocol, plasmid DNA was rescued from individual colonies using the Zyppy kit (Zymo Research), and introduction of the desired mutations comprising pESO-H7sdm (Fig 1A) was verified with bi-directional sequencing using the primers BTSeqF 5’-CTGCTCCGAACAATAAAGATTCTAC-3’ and BTSeqR 5’-GTATGTGTAAAGTTGGTAACGGAAC-3’.

Next, a DNA cassette containing the histidine-saturated CDRH1 was fabricated from a 90bp oligonucleotide mix and two complementary oligonucleotide primers. The 90bp mixed oligonucleotide DNA (purchased from IDT) was designed using degenerate bases, allowing the possibility of histidine at every codon in CDRH1, while maintaining 30bp homology to wild-type scFv H7 at both the 5’ and 3’ ends (IP90T 5’-AGGTCCCTGAGACTCTCCTGTGCAGCCTCTCRWYWCMMCYWCMRTMRCYATSMTMWKCAC TGGGTCCGCCAGGCTCCAGGCAAGGGGCTG-3’, where S = G/C, R = A/G, Y = C/T, K = G/T, M = A/C, W = A/T). IP90T was made double-stranded and extended to 100bp via 5 cycles of standard PCR with OneTaq 2X GC Master Mix (NEB) and a single primer IPCDH1ampF 5’-CCACCCACTCCAGCCCCTTGCCTGGAGC-3’ (Fig 1A). The size and homogeneity of the product were verified by agarose gel electrophoresis. A DNA Clean and Concentrate 5 kit (Zymo Research) was used to purify the 100bp PCR product. The final mutagenic DNA cassette was produced by a second PCR reaction with OneTaq 2X GC Master Mix using the first PCR product as a template and the primers IPCDH1ampF and IPCDH1ampR 5’-CCAGCCTGGGAGGTCCCTGAGACTCTCCTGTGC-3’. The resulting 120bp double-stranded cassette was separated by agarose gel electrophoresis and purified with the Zymoclean kit (Zymo Research).

Finally, The H7CDRH1his library was created by electroporation and homologous recombination of the histidine-saturated 120bp mutagenic DNA cassette, and the (SpeI) linearized pESO-H7sdm acceptor vector in yeast [34,35] (Fig 1A). Library size was determined by plating serial dilutions and colony counting. A dozen yeast colonies were selected at random and plasmid DNA was rescued with the Zymoprep II kit (Zymo Research). The type (histidine or non-histidine) and distribution of mutations in the scFv H7 were ascertained by sequencing as described above. All mutations were found confined to the CDRH1 (Table 1).

**Table 1:** CDRH1 amino acid sequences of CDRH1his clone subsets.

scFv H7 wild type CDRH1^a^	R	F	T	F	S	S	Y	A	M	H
	28	29	30	31	32	33	34	35	36	37
Randomly selected clones from CDRH1his	17%	17%	17%	17%	8%	25%	33%	25%	25%	
LD1	R	F	**P**	**L**	S	**H**	**H**	**D**	**I**	H
LD2	R	F	**H**	**H**	S	**R**	Y	**D**	M	H
LD3	R	**Y**	T	F	**H**	**R**	Y	A	**K**	H
LD4	R	**Y**	**N**	**Y**	**R**	**R**	Y	**D**	**I**	H
LD5	**Q**	F	T	**Y**	**N**	**R**	**H**	**P**	M	H
LD6	**H**	Y	**H**	L	S	S	Y	**H**	L	H
LD7	R	F	**N**	**L**	**N**	**H**	**H**	**H**	**H**	H
LD8	**H**	**H**	**N**	**H**	**R**	**N**	Y	A	**N**	H
LD9	R	**Y**	**P**	F	S	S	Y	**H**	**L**	H
LD10	R	F	T	**Y**	**N**	S	Y	A	**K**	H
LD11	R	F	T	**T**	S	**H**	**H**	**P**	**H**	H
LD12	R	**H**	T	F	S	S	Y	A	**H**	H
CDRH1 mutants with increased dissociation at pH5.5	25%	0%	25%	25%	63%	63%	75%	13%	13%	
M4	R	**L**	**N**	**Y**	**N**	S	**H**	**H**	M	H
M8	**H**	**Y**	**N**	**Y**	S	**N**	**Y**	**P**	M	H
M10	**H**	**L**	**H**	**H**	**N**	**H**	**H**	**P**	**L**	H
M11	R	**L**	**N**	F	**H**	**H**	**H**	A	M	H
M16	R	**Y**	**P**	F	**H**	**H**	**H**	**D**	**H**	H
M17	R	F	**H**	**H**	**H**	**R**	**Y**	A	**Q**	H
M20	R	F	**P**	F	**H**	**H**	**H**	**P**	**I**	H
M23	R	F	**S**	F	**H**	**H**	**H**	**P**	**I**	H
CDRH1 mutants with reduced dissociation at pH5.5	0%	13%	38%	0%	0%	13%	0%	25%	13%	
N4	R	F	**H**	F	**N**	**R**	Y	**H**	M	H
N5	R	F	T	F	**N**	**N**	Y	A	M	H
N7	R	**H**	**H**	**L**	S	S	Y	**D**	M	H
N10	R	F	**H**	**L**	**N**	S	Y	**D**	M	H
5P4	**Q**	F	**H**	F	**N**	**N**	Y	**D**	**L**	H
5P7	R	F	T	F	S	**H**	Y	**D**	**L**	H
5P9	R	F	T	F	S	**N**	Y	**H**	**H**	H
5P15	**Q**	**H**	**N**	**L**	**R**	S	Y	**H**	**L**	H

^a^The wild-type H7 CDRH1 is shown across the top with heavy chain residue numbers above (Kabat numbering). Percentages at the top of every section indicate the incidence of histidine at that position for the clones in the table. Bold font indicates a mutation from wild-type and bold-underline font indicates histidine.

### FACS-based screening and selection of pH-sensitive scFv

Yeast for surface display were grown in SD-CAA at 30°C, 260 rpm overnight. The following day, cultures were diluted to an OD600nm of 0.3 and grown until the OD600nm reached 1.0. Induction of surface display was accomplished by replacing SD-CAA with an equivalent volume of SG-CAA and incubating at 20°C, 260 rpm for 16–20 h. Finally, induced yeast were washed three times in ice-cold 100 mM phosphate buffer pH 7.4, containing 50 mM NaCl and 1 g/L bovine serum albumin (PBSA). Unless specifically noted, PBSA was the default buffer used for all washing, dilution, and resuspension steps.

The CDRH1his library contained approximately 3 x 10^7^ members and was initially enriched for TfR binders at neutral pH to ensure scFv retained wild-type functionality prior to imposing pH 5.5 selection pressure. For the first two rounds or sorting, 1 x 10^8^ induced yeast cells were incubated with 2mL 50nM recombinant human TfR (rhTfR, soluble extracellular domain, R&D systems) to saturate scFv binding. After rotating at room temperature for two hours, yeast were washed twice with PBSA to remove non-specifically bound rhTfR. In parallel, 2 x 10^6^ control yeast (H7 or D1.3) were incubated in 100 μL of 50 nM rhTfR. After washing, yeast were prepared for FACS; all subsequent washing and immunolabeling steps were carried out at 4°C to prevent further antigen dissociation. Full-length expression was detected using a rabbit polyclonal antibody against the *c-myc* epitope tag (PA1-24484, Thermo-Fisher, diluted 1:1000), followed by goat an anti-rabbit Alexa 488-conjugated secondary antibody (A-11008, Life Technologies, diluted 1:500). TfR-binding was detected by a mouse monoclonal anti-hTfR antibody (R&D Systems, clone 29806, diluted 1:100) followed by a goat anti-mouse Alexa647-conjugated secondary antibody (A-21235, Invitrogen, diluted 1:500). Yeast libraries were sorted in purity mode at approximately 1 x 10^7^ events per hour on a FACSAriaII (Becton-Dickinson).

Following two rounds of enrichment pH 7.4 binding, two rounds of sorting were carried out that included a pH 5.5 incubation step to isolate pH-sensitive binders. 1 x 10^7^ induced, washed yeast were incubated at room temperature for 2 h with 500 μL of 50 nM rhTfR diluted in PBSA. The yeast were pelleted by centrifugation and the rhTfR solution was aspirated. Yeast were resuspended in 1 mL 200 mM pH 5.5 sodium MES containing 1 g/L BSA (MBSA) and rotated at room temperature for 10 min. The reaction was quenched by adding 4 mL ice-cold PBSA and immediately pelleting the yeast. Two additional washes in ice-cold PBSA were carried out, prior to labeling the yeast for FACS as described above. 2 x 10^6^ of each control yeast (H7 and D1.3) were prepared identically, both with and without the pH 5.5 shift, to serve as experimental and gating controls. Pools from the selection process above were plated on SD-CAA at a 1:10,000 dilution. Single colonies were selected and subjected to the same pH-shift and FACS labeling steps described above. Geometric mean fluorescence intensities of the antigen binding populations (MFI, background fluorescence from scFvD1.3-displaying yeast subtracted) were quantified with the FlowJo (Tree Star). Fraction rhTfR bound was calculated by dividing the MFI at pH 5.5 by MFI pH7.4.

### Apparent equilibrium binding affinity of scFv on yeast surface

Serial dilutions of rhTfR ranging from 2 pM– 50 nM were prepared in PBSA, at sufficient volume to avoid ligand-depleting conditions. ScFvs M16, N5, H7, and D1.3 were freshly transformed by the LiAc/ssDNA/PEG method [36] into the appropriate yeast strains for surface display. Yeast were grown and induced as above; 5 x 10^5^ induced yeast displaying a given scFv clone, were collected and washed twice in PBSA. Washed yeast were incubated with rhTfR dilutions, shaking in a 20°C incubator for 3 h. Expression and TfR binding were analyzed by flow cytometry (FACSCalibur, Becton-Dickinson) using the same primary and secondary antibodies described above, collecting at least 15,000 cell events per sample. Fraction of rhTfR bound (with background due to non-specific binding by negative control scFv D1.3 subtracted), was quantified using fluorescence data (MFI) from the A647 (anti-TfR) channel. Fraction bound values were fit to a bimolecular equilibrium binding model to determine the apparent equilibrium binding constant (K_d_).

### Secretion and purification of soluble scFv

Soluble scFv were secreted and purified from YVH10 supernatants as detailed previously [33]. Briefly, scFvs were subcloned into the pRS316-GAL-4420 expression vector and transformed into YVH10. After 72 h growth in SD-2XSCAA and 72 h induction in SG-2XSCAA at 20°C, supernatants were collected, sterile-filtered, dialyzed into TRIS-buffered saline (TBS, 25 mM Tris, 150 mM NaCl, 2 mM KCl, pH 7.9) and scFv were purified using Ni-NTA Superflow resin (Qiagen). Purified protein was dialyzed in Dulbecco’s PBS (2.68 mM KCl, 136.89 mM NaCl, 8.10 mM Na2HPO4, 1.47 mM KH2PO4, Sigma-Aldrich), 0.2 uM filtered, and stored at 4°C. ScFv purity was analyzed by SDS-PAGE and Western blotting, while protein concentration was measured using the Bradford Reagent (Sigma-Aldrich). ScFv D1.3, was secreted, purified, and employed as a negative control.

### Binding and pH-sensitive dissociation of rhTFR from soluble scFvs

To assay for specific activity of the purified scFv, streptavidin-coated Dynabeads Biotin-Binder paramagnetic particles (~2.8 μm diameter, Life Technologies) at a concentration of 1 x 10^7^ particles/mL, were incubated with biotinylated mouse anti-c-*myc* MAb (clone 9E10, 05–419, EMD Millipore, diluted 1:250) in TRIS-buffered saline pH 7.4 containing 1 g/L BSA (TBSA) overnight, at room temperature. 9E10 MAb-coated particles were washed three times in TBSA, divided into aliquots (1 x 10^5^ particles each), and dispensed into 96-well polypropylene plates. Subsequently, scFvs were captured on the particles through their carboxy-terminal c-*myc* epitope. To accomplish scFv capture, 200 μL of purified scFv at a concentration of 100 nM (an amount known to saturate available 9E10 on the bead surface) was added to each experimental well and allowed to equilibrate for 2 h. The particles were washed once with TBSA, resuspended in 50 uL of 50 nM rhTfR and equilibrated for 1 h. The particles were collected magnetically and the rhTfR solution was aspirated. Particles were resuspended in 200 μL MBSA, pH 5.5 and incubated at room temperature for 10 min. Alternately, particles were resuspended in 200 μL TBSA, pH 7.4 to serve as neutral pH dissociation controls. All reactions were quenched by adding 300 μL ice-cold TBSA. Fluorescent labeling was completed using ice-cold reagents with the plate resting on ice. Two additional washes in TBSA were carried out, prior to labeling the particles for flow cytometry. Particle-bound scFv was detected using a DyLight650-conjugated anti-his6 antibody (Abcam, ab117504, diluted 1:300 in TBSA). rhTfR binding was detected by mouse anti-hTfR PE conjugate (FAB2474P, R&D systems, diluted 1:10 in TBSA). MFIs of the labeled beads were quantified using FlowJo; the bound rhTfR per scFv on the bead was determined as the ratio of PE fluorescence (TfR binding) divided by the DyLight650 fluorescence (scFv amount). Specific, pH-driven dissociation of rhTfR was then calculated by dividing the bound rhTfR per scFv at pH 5.5 by that at pH 7.4.

### Immunocytochemistry

SKBR-3 cells were seeded on sterile 12mm poly-D-lysine coated glass coverslips (BioCoat, Becton-Dickinson), housed in a 24-well plate, at 2 x 10^5^ cells per well. Cells were cultured in 1mL SKBR3GM for 24–72 h. prior to experimentation. Growth medium was aspirated, cells were washed once with PBS containing 0.9mM calcium chloride and 0.49 mM magnesium chloride (PBSCM, Sigma-Aldrich), then 1.0 mL SFM containing 10 μM deferoxamine mesylate iron chelator (Sigma-Aldrich) was added. Cells were incubated at 37°C / 5% CO_2_ for 15 min to starve them of iron. Depending on the experimental goals, artificial dimers (ADs) or monomeric scFvs were used. ADs were created by pre-incubating purified scFv having c-*myc* epitope with either unconjugated or Alexa488-conjugated anti-c-*myc* antibody (clone 9E10, Covance or clone 9E10-A488, 16–308, EMD Millipore) at a 4:1 molar ratio in PBS containing 10% goat serum (PBS10G) as previously described [37]. After a 1 h. incubation at room temperature, ADs were diluted to 200 nM concentration in serum-free media For immunocytochemistry, ADs or scFv at 200 nM (300 μL total volume) were dosed onto live cells (iron-starved as above) and allowed to traffic for 2 h. at 37°C / 5% CO_2_. After trafficking the cells were washed with PBSCM and fixed in 3.7% PFA, 4% sucrose for 15 min at room temperature. Fixation was quenched by rinsing with DPBSCM containing 50 mM ammonium chloride. Cells were blocked with PBS10G containing 1% BSA for 30 min at room temperature. Antibody incubations were all 1 h at room temperature, and antibodies were diluted in PBSA. If intracellular antigens were to be stained, 0.1% saponin (Sigma) was added to the blocking and antibody dilution buffers. Primary antibodies used were anti-LAMP1 (Rabbit pAb, ab24170, Abcam, diluted 1:300), anti-EEA1 (Rabbit mAb, C45B10, Cell-Signaling, diluted 1:200), anti-LAMP2 (Mouse mAb, ab25631, diluted 1:300), and 9E10 (05–419, Millipore, 1:250). Secondary antibodies were all host species specific, and Alexa555 conjugated (Life Technologies, diluted 1:250). DAPI (Life Technologies) was used at 300 nM to visualize cell nuclei. Images were captured on an AxioImager-Z2 with a 63x oil-immersion objective (Zeiss). Meta z-stacks where created by automated fine, focal adjustment. Slice width was set to 1 μm, 10 slices were captured, and then reconstructed as a maximum intensity z-projection using ImageJ (v1.43u, NIH). Pearson’s co-localization coefficients were calculated from N ≥ 2 independent, background subtracted images comprising 20–40 cells each using ImageJ (Fiji, www.fiji.sc) with the Coloc 2 plugin.

A variation of the immunocytochemistry procedure above was used to differentially visualize internalized versus surface-bound scFv. Since monomeric and artificially dimerized scFvs yielded indistinguishable intracellular distributions, AD’s made with unconjugated anti-c-*myc* antibody were used for these experiments. After trafficking, cells were washed three times in ice cold PBS, fixed for 30 min in ice-cold PFA. Cells were blocked for 30 min at room temperature, and then incubated with anti-mouse-Alexa647 (diluted 1:500) for 30 min at room temperature to label extracellular scFv. Next, the cells were permeabilized for 10 min. with PBS containing 0.2% Triton X-100. To visualize intracellular scFv dimers, permeabilized cells were incubated for 30 min. at room temperature with anti-mouse-Alexa488 (diluted 1:500) which had been diluted in PBS10G containing 0.1% Triton X-100.

### Flow cytometric quantification of cell-associated scFv

SK-BR-3 were seeded at a density of 1 x 10^5^ cells per well in 48 well TC-treated plates and grown 48 h. in advance for flow cytometry. Growth medium was aspirated, cells were washed once with PBCSM then 0.5 mL SFM containing 10 μM deferoxamine mesylate iron chelator (Sigma-Aldrich) was added. Cells were incubated at 37°C / 5% CO_2_ for 15 min to starve them of iron. After iron starvation, cells were washed once in PBSCM and 125 μL of 200 nM AD were added. Cells were incubated at 37°C / 5% CO_2_ for 2 h to allow the ADs to traffic. For experiments involving inhibition of intracellular acidification, 200nM of bafilomycin A1 (BafA1) (Santa Cruz) was added in SFM and incubated with cells for 45 min, prior to the iron chelation step. The reminder of the experimental steps were identical to those denoted above with the addition of 200nM BafA1 to each solution. Following trafficking, the AD solution was aspirated and the cells were washed twice with PBSCM. For quantification of total-cell associated antibody, cells were detached by incubation with non-enzymatic cell dissociation buffer (Gibco). To quantify the internalized antibody fraction only, extracellular protein degradation and cell detachment were achieved simultaneously by incubation with 0.25% Trypsin-EDTA solution (Gibco). In either case, detached cells were transferred into 3 mL of ice-cold DPBS10G and immediately centrifuged at 180 x g for 5 min. Cells were resuspended in 300 μL fixation buffer (3.7% PFA, 4% sucrose) and incubated for 10 min at room temperature before being quenched with 3 mL PBS containing 50 mM ammonium chloride and centrifuged once more at 180 x g for 5 min. Cells were resuspended for flow cytometry in 500 μL PBS containing 20 g/L BSA and 2 mM EDTA. Cells of normal size and morphology were gated and the MFI in the Alexa488 channel was quantified using FlowJo. MFI data from trypsinized cells corresponded to the internalized fraction of scFv, while data from non-enzymatically detached cells corresponded to total cell-associated scFv. Surface fraction was calculated by subtracting the internalized signal from the total signal. Non-specific background signal equal to the MFI of irrelevant scFv-treated cells was subtracted from all samples.

### Statistics

For all calculations in this manuscript, data from multiple independent experiments on each of a minimum of two days were collectively used to determine quantitative parameters, their associated 95% confidence intervals (95% CI), and significance levels (p-value) by student’s t-test. The resultant total number of independent experiments (n-value) for each assay is denoted in each figure legend.

## Results

### Assessment of scFv H7 pH-sensitivity and histidine mutagenesis

**Fig 2:**
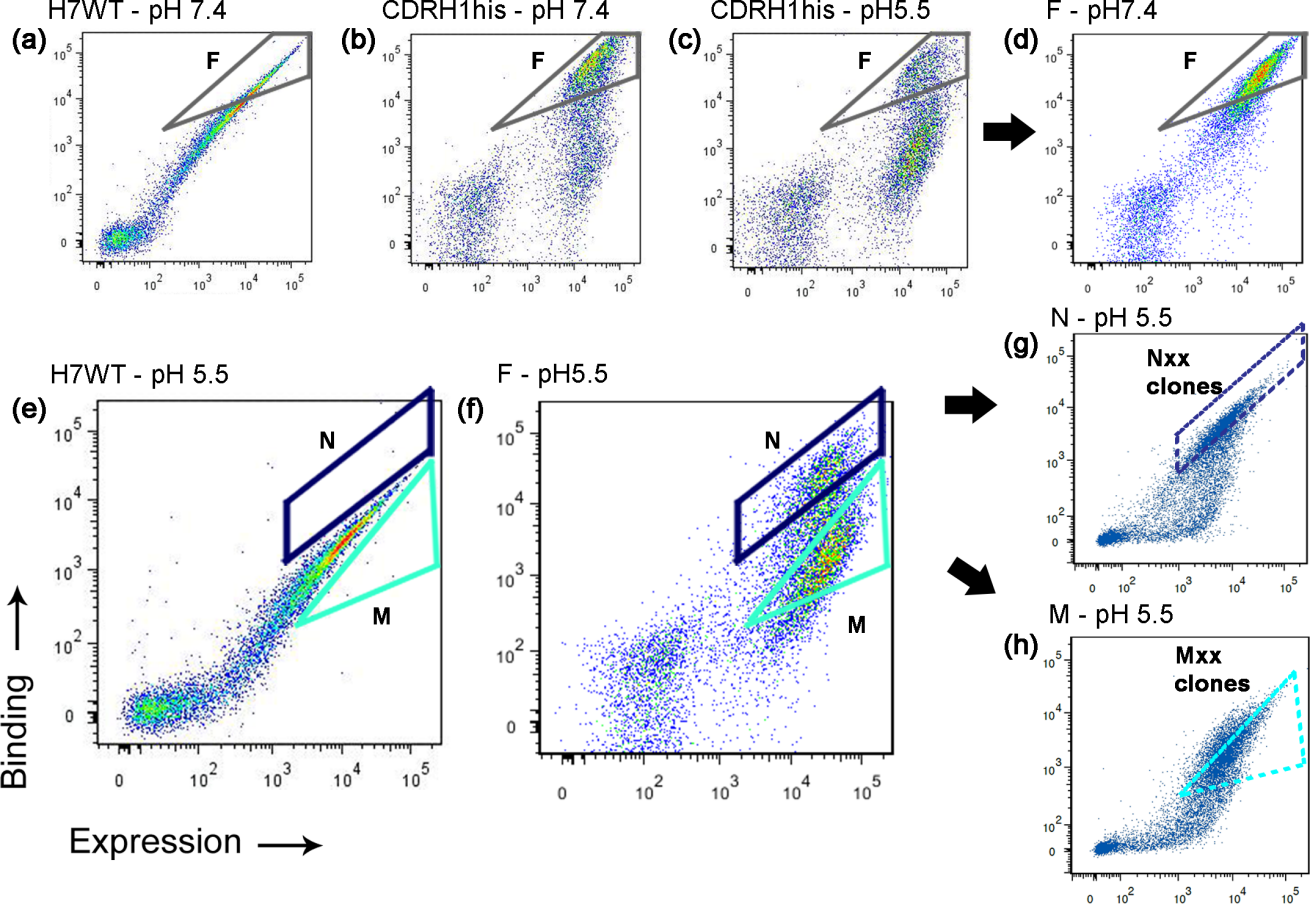
Flow-cytometric screening of the CDRH1his library for scFvs having affected dissociation from TfR at pH 5.5. Dot plots depict the behavior of the various scFv populations after saturation with rhTfR followed by a 10 minute dissociation step in pH 5.5 or 7.4 buffers. TfR-binding is indicated on the *y*-axis while scFv surface expression level is indicated on the *x*-axis. Sample gates are drawn only for illustrative purposes for the reader to follow the sorting enrichment procedure. (a) Antigen binding and expression of wild-type H7 at pH 7.4. (b and d) Pool F was derived from the CDRH1his library via two rounds of sorting at pH 7.4 and comprises neutral pH TfR-binders. (c and also S1 Fig) A population comprising pH-insensitive binders exists in CDRH1his and can be visualized in gate F after the library is subject to pH 5.5 dissociation. (e) Antigen binding and expression of wild-type H7 at pH 5.5. (f) Both pools M and N were obtained by selecting for different populations within pool F, post dissociation at pH 5.5. (f and g) Gate N was placed near the maximum in TfR-binding signal to select for pH-insensitive scFvs and ultimately yielded pool N. (f and h) Alternatively, gate M was placed just above the no-antigen control, but below gate N to select for the pH-sensitive binders ultimately found in pool M (see also S1 Fig). (g and h) Flow-cytometric analysis of the M and N pools. Individual clones arising from these screening strategies are listed in Table 1 and quantitative clonal dissociation behavior is shown in Fig 3. Further detailed explanation of the screening strategy and pools depicted in the various panels can be found in the text.

**Fig 3:**
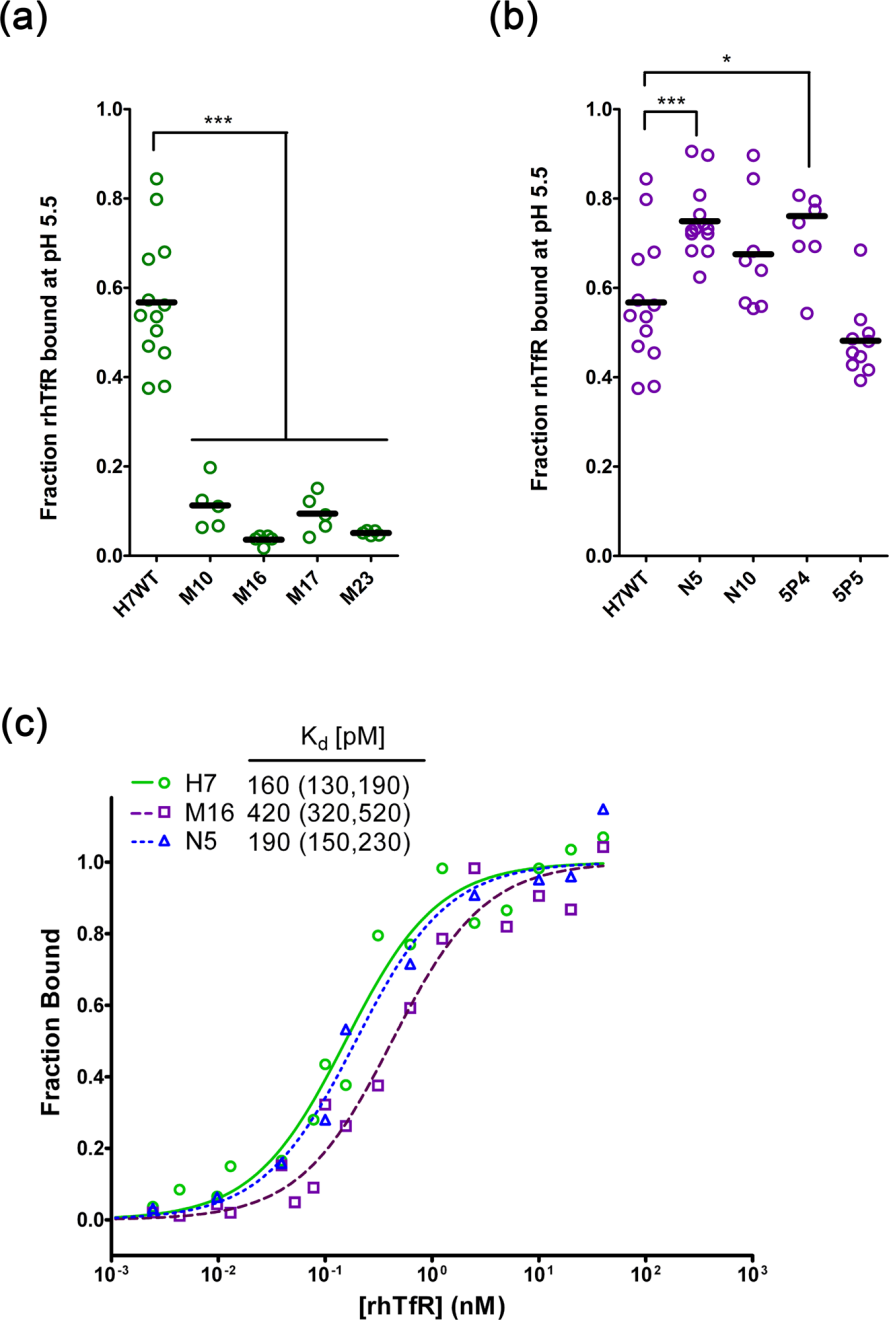
Quantitative analysis of scFvs isolated from the CDRH1his library using yeast surface display. (a) Fraction TfR bound to M mutants after 10 minute incubation at pH 5.5. (n = 5 for M mutants and n = 13 for H7, and ***, p <0.001) (b) Fraction TfR bound to N and 5P mutants after 10 minute incubation at pH 5.5. (n = 8 for N and 5P mutants and n = 13 for H7 mutant as in panel a) and ***, p <0.001 *, p <0.05). (c) Apparent equilibrium binding affinity of select clones on the surface of yeast at pH 7.4. Mean data from five independent experiments are plotted along with the fitted equilibrium binding isotherms. The legend shows numeric values for the best-fit equilibrium binding affinity (K_d_) and associated 95% CI.

As a first step in engineering pH-sensitive TfR binding, the intrinsic pH-sensitivity of wild-type anti-human TfR scFv H7 was established using a yeast surface display dissociation assay (Fig 1B). After saturation of yeast-displayed H7 with recombinant human TfR (rhTfR) at neutral pH, yeast were exchanged into acidic buffer (pH 4.0–6.5) for 10 minutes to allow for TfR dissociation, and the fraction of TfR remaining bound was assessed by flow cytometry. H7 was found to dissociate from TfR in a pH-dependent manner, particularly at pH 5.0 and below where TfR binding was almost completely abolished (Fig 1B, histogram). However, at pH 5.5, TfR binding was decreased by just 44% compared to the level observed at pH 7.4 (Figs 1B, 2 and 3A). Thus, there was a clear opportunity for engineering scFvs having a more rapid (<10 min) dissociation from TfR at pH 5.5, conditions chosen based on their relevance to endogenous transferrin-TfR trafficking [19,20,38].

Previously, a number of scFv H7 variants were isolated possessing improved equilibrium binding affinity at neutral pH [33]. Sequence analysis of randomly selected clones from the affinity-matured pools revealed a strong bias (20 out of 27 unique mutations) toward amino acid changes in the heavy chain, including CDRH1 and CDRH2 [33], suggesting a direct role for each of these two CDRs in TfR binding. However, amongst mutations affecting the most substantial affinity enhancement, those located in CDRH2 predominated, indicating a comparatively important role for CDRH2 in mediating TfR binding at neutral pH. Thus, CDRH1 was chosen as a locus for engineering an scFv with increased dissociation from TfR at pH 5.5. Given that multiple histidine substitutions in combination have been shown to be particularly effective at imparting pH-sensitive antigen binding [39], a recombinant library was designed to saturate CDRH1 with all combinations of histidine, from a single histidine at each position to ten consecutive histidines (Fig 1A and Materials and Methods). The resulting H7 CDRH1his yeast display library comprised 3 x 10^7^ clones and substantially oversampled the theoretical diversity (1.8 x 10^5^) arising from designed nucleotide degeneracy. Sequencing of 12 randomly selected clones from the unselected library indicated a rich mix of histidine mutations in CDRH1, both in number and position (Table 1).

### Screening the CDRH1his library

The CDRH1his library was screened by TfR saturation of surface-displayed scFvs followed by 10 minutes of TfR dissociation at pH 5.5 (Fig 1B). Evaluating the response (full length-display and antigen binding) of the starting library to this treatment yielded two key observations. First, at pH 7.4, approximately 40% of the library clones retained substantial TfR binding despite mutations to the CDRH1 loop (Fig 2B, gate F). Second, when incubated at pH 5.5 for 10 minutes, many of the clones dissociated from TfR as desired, but a sizeable population (~20%) remained bound (Fig 2C, gate F). H7 was comparatively less responsive to pH 5.5 treatment (Fig 2E and S1 Fig). Given these responses, we proceeded to screen for both pH-insensitive and pH-sensitive mutants.

Since it was desired to maintain anti-TfR potency at neutral pH, two initial rounds of FACS-based sorting were performed at pH 7.4 to purify TfR binders from the CDRH1his library (Fig 2B, gate F). The resulting functional pool, “F”, was essentially free from scFv mutants with impaired TfR binding at neutral pH (Fig 2D), while still containing scFvs capable of the desired TfR dissociation at pH 5.5 (Fig 2F). Subsequently, to isolate pH-sensitive TfR binders, pool F was sorted using gate “M”, bracketed on the high end by the H7, pH5.5 TfR binding signal, and on the low end by the no-antigen control (Fig 2E and 2F and S1 Fig). Two rounds of FACS sorting in this fashion, yielded pool “M”, a population with significantly attenuated TfR binding after 10 minutes at pH5.5 (Fig 2H). In parallel, a second non-overlapping gate, N, was added above the M gate. Clones in pool “N” exhibited increased retention of TfR binding compared to H7 at pH 5.5 (Fig 2G and S1 Fig). In contrast to the pH-sensitive M pool, clones in the N pool maintained strong associated with TfR at pH 5.5 and represented a class of pH-insensitive, TfR binding, scFvs. Finally, pH-insensitive binders were isolated directly from the CDRH1his library without going through the F pool as an intermediate (S1 Fig). These clones possessed properties similar to pool N and were referred to as 5P.

### Clonal scFv analysis from M, N, and 5P pools

A combined 92 clones from the M, N, and 5P pools were screened for TfR binding at pH 7.4 and extent of dissociation after 10 minutes at pH 5.5. Those clones exhibiting the desired pH sensitivity (M) or insensitivity (N and 5P) were sequenced to evaluate histidine substitution in the engineered CDRH1s. The pattern of histidine substitution in unique clones from pH-sensitive pool M was distinct from that found in the N and 5P clones with a strong bias toward histidine in at least three of the central positions (V_H_31-V_H_34) of CDRH1 (Table 1). Additionally, this central histidine core was accompanied by the mutation of at least one of the adjacent residues (V_H_30 or V_H_35) to proline in the majority of the M clones (e.g. Table 1, clones M8 and M16). In contrast, none of the unique clones analyzed from the pH-insenstive N or 5P pools contained either of these features. Instead, the wild-type serines at V_H_32 and V_H_33 were often mutated to arginine or asparagine, with any newly added histidines appearing away from the V_H_31-V_H_34 “core” at either V_H_30 or V_H_35 (e.g. clones N4 and 5P4 in Table 1C). N and 5P series clones also had, on average, fewer total mutations than their M series counterparts (37% versus 64%), and a wild-type tyrosine at position V_H_34 that remained invariant.

Based on these general mutational classes, four pH-sensitive (M10, M16, M17, and M23) and four pH-insensitive (N5, N10, 5P4, and 5P15) mutants were quantitatively assayed for fractional TfR dissociation at pH 5.5. For reference, wild-type scFv H7 retained 56% of bound TfR after the 10 minutes pH 5.5 incubation (Fig 3A). All of the M clones exhibited greater than 90% dissociation in this same timeframe (Fig 3A, p < 0.001). Two of the N and 5P clones exhibited some modest reduction in association; clones N5 (75 ± 5% bound, p < 0.001) and 5P4 (76 ± 11% bound, p < 0.05) retained roughly 25% more bound TfR during low pH incubation than H7 (Fig 3B).

Due to the significant number of mutations in CDRH1 (Table 1), we measured the apparent equilibrium binding affinity at pH 7.4 for the leading pH-insensitive (N5) and pH-sensitive (M16) clones. As shown in Fig 3C, N5 exhibited a statistically equivalent apparent equilibrium binding affinity (190 ± 40 pM) compared to H7 (160 ± 30 pM). M16 possessed a 2.5-fold attenuated equilibrium binding affinity (420 ± 100 pM). Thus, the apparent affinities of M16 and N5 both remained sub-nanomolar although CDRH1 was substantially mutated

### Characterization of pH-dependent scFvs

**Fig 4:**
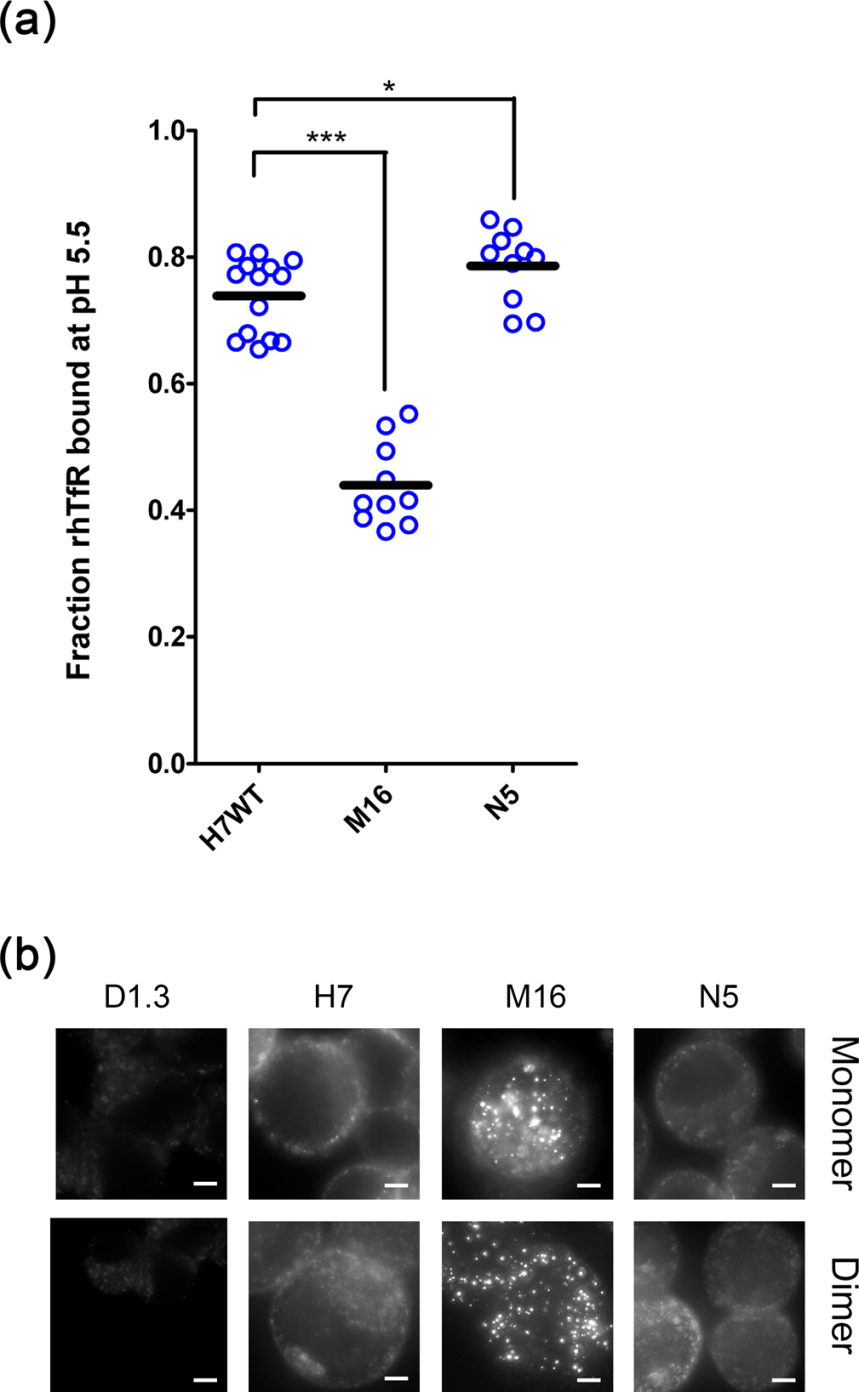
Analysis of soluble M16, N5 and H7 scFvs. (a) Magnetic bead assay to determine the pH-sensitivity of TfR-binding using soluble protein. Soluble scFvs were captured on the bead surface via their *c-myc* epitope tags and incubated with rhTfR. After 10 minute incubation in pH 7.4 or pH 5.5 buffer, the fraction of TfR bound at pH 5.5 versus pH 7.4 was assayed by flow cytometry. (n = 10 for M16, n = 14 for H7 and n = 10 for N5, ***, p <0.001, *, p <0.05) (b) Whole-cell immunolabeling using monomeric scFvs and artificial scFv dimers formed via the scFv *c-myc* epitopes as described in Materials and Methods. Proteins were allowed to traffic for 2 hours in SK-BR-3 cells at 37°C. Meta-z stacks were captured and recombined into a maximum intensity z-projection to better visualize surface versus intracellular protein. Scale bars = 5μm.

The 16 scFvs from M, N, and 5P pools listed in Table 1 were secreted from yeast as soluble scFvs. Of these, only clones M16 and N5 could be expressed and purified in amounts sufficient to allow further characterization, indicating that certain CDRH1 mutations, alone or in concert, could negatively impact scFv secretion. Maintenance of pH-sensitive and pH-insensitive TfR binding was evaluated for scFvs M16 and N5, respectively, through capture of soluble antibody onto rhTfR-coated beads followed by low-pH dissociation and flow cytometric analysis. The rank ordering of pH sensitivity was consistent with that observed in the yeast surface display format, although less overall dissociation from rhTfR was observed using this assay. ScFv M16, retained 44 ± 5% of TfR binding after 10 minutes of pH 5.5 incubation versus 74 ± 3% for WT H7 (Fig 4A, p < 0.001), while the pH-insensitive clone, scFv N5, retained slightly more TfR-binding than H7 (Fig 4A, 80 ± 4% p < 0.05).

**Fig 5:**
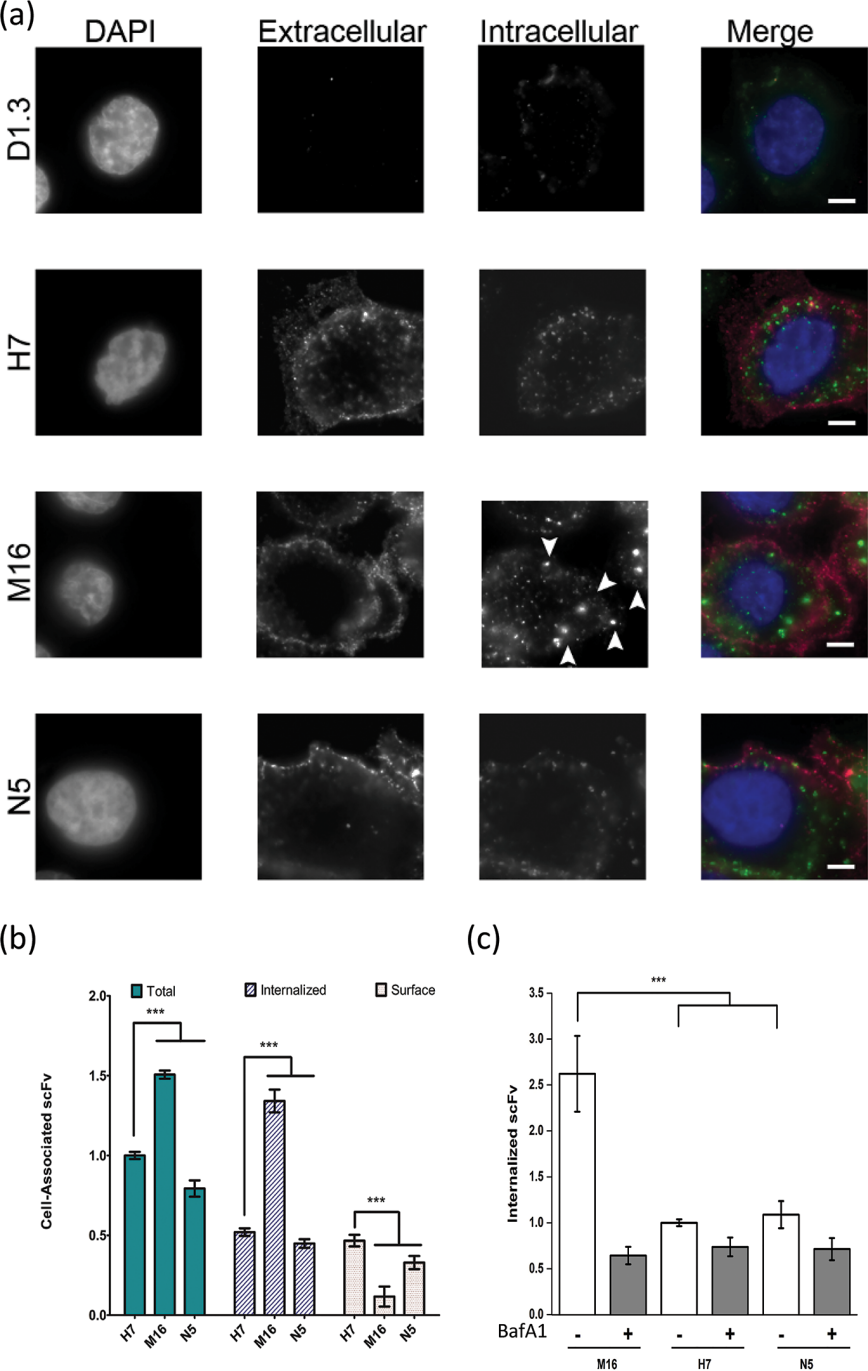
Endocytosis of scFvs into SK-BR-3 cells and quantification of intracellular accumulation. (a) Immunolabeling of surface and internalized scFvs. Soluble scFvs were dimerized via their *c-myc* epitope tags and pulsed onto SK-BR-3 cells at 37°C for 2 hours to allow for internalization. Fluorophores with different emission spectra were used to immunolabel surface-bound scFv (Alexa647, pseudo-colored pink) and, after permeabilization, intracellular scFv (Alexa488, pseudo-colored green). Nuclei were visualized with DAPI (pseudo-colored blue). Arrowheads indicate the distinct pattern of internalized scFv M16. Scale bar is 5μm. (b) Quantification of scFv association with SK-BR-3 cells. scFv pre-dimerized with 9E10-Alexa488 was dosed onto live SK-BR-3 cells and allowed to traffic at 37°C for 2 hours (see Materials and Methods for assay details) and total cell-associated scFv assayed by flow cytometry. Internalized scFv was also quantified by flow cytometry after removal of the cell surface bound scFv by trypsinization. Total cell-associated scFv is normalized to H7 scFv and internalized and surface scFv sum to the totals for each clone. (n = 8 for H7 and M16 and n = 12 for N5, ***, p < 0.001). (c) Quantification of scFv internalization after pre-treatment with endosomal acidification inhibitor, BafA1. Internalized scFv was normalized to that for wild-type H7 in the absence of BafA1 treatment (n = 6 for H7, M16 and N5, ***p<0.001).

Given that the TfR dissociation behavior of M16 and N5 was distinct from wild-type H7, both in surface-displayed and soluble forms, we next explored whether pH-sensitive TfR binding could alter intracellular antibody trafficking. When applied to the human breast cancer cell line (SK-BR-3), scFvs bound to the cell surface and were internalized. Monomeric scFvs were dosed onto SK-BR-3 cells and incubated at 37°C for 2 hours to allow for binding and internalization. H7 and N5 tended to bind the periphery of cells, with some punctate localization just inside the cell membrane compared to M16 which had reduced surface labeling in favor of distinct, intracellular structures with qualitatively elevated concentrations of scFv (Fig 4B). If scFvs were first pre-dimerized, a similar distribution was observed (Fig 4B). A two fluorophore immunolabeling procedure was employed to unequivocally demonstrate binding and internalization of M16, N5 and H7. As suggested by the single color immunolabeling (Fig 4B), binding and internalization were both clearly observed for each scFv (Fig 5A). The intracellular staining for scFv M16 was unique, however, showing a significant number of large, punctate, vesicular structures, while H7 and N5 yielded smaller membrane localized structures (Fig 5A).

Next, the effects of pH-sensitive TfR binding on the total cell-association and endocytosis of the engineered scFvs were quantitatively assessed by flow cytometry. Compared to H7, N5 had, on average, 20% less total cellular association (Fig 5B, p <0.001), while the levels of total cellular association for scFv M16 were 1.5-fold higher (Fig 5B, p <0.001). To directly quantify the internalized fraction, we capitalized on the fact that TfR contains a membrane-proximal trypsin cleavage site which can be used to remove surface-exposed TfR along with any scFv that might be bound [40]. When trypsinized cell samples were assayed by flow cytometry the levels of internalized scFv M16 were 2.6-fold greater than wild-type H7 (Fig 5B, p <0.001) as a result of more total cellular association (1.5-fold) and a higher fraction of total cell-associated scFv being found internally (87%). N5 and H7 on the other hand, were much more similar in terms of percentage of total cell-associated scFv that was internalized (Fig 5B, 52% of total H7 was internalized, and 56% of total N5 was internalized). Taken together, these data confirmed the enhanced internalization of M16 compared with N5 and H7, as previously suggested qualitatively by the immunocytochemistry images. In order to examine whether pH-dependent phenomena were driving the difference in increased scFv M16 internalization, the amount of internalized scFv was monitored after treatment with bafilomycin A1 (BafA1), an inhibitor of endosomal-lysosomal acidification [41,42]. In contrast with the untreated, control samples where scFv M16 internalized at levels 2.5-fold greater than wild-type H7 (Fig 5C, p<0.001), treatment with BafA1 completely removed the beneficial effects of M16 on internalization such that it showed no difference in internalization in comparison to wild-type H7 or scFv N5 (Fig 5C, p>0.05). These results indicate that endosomal acidification is required to observe the beneficial internalization properties of M16, confirming the important role of pH-sensitive binding.

**Fig 6:**
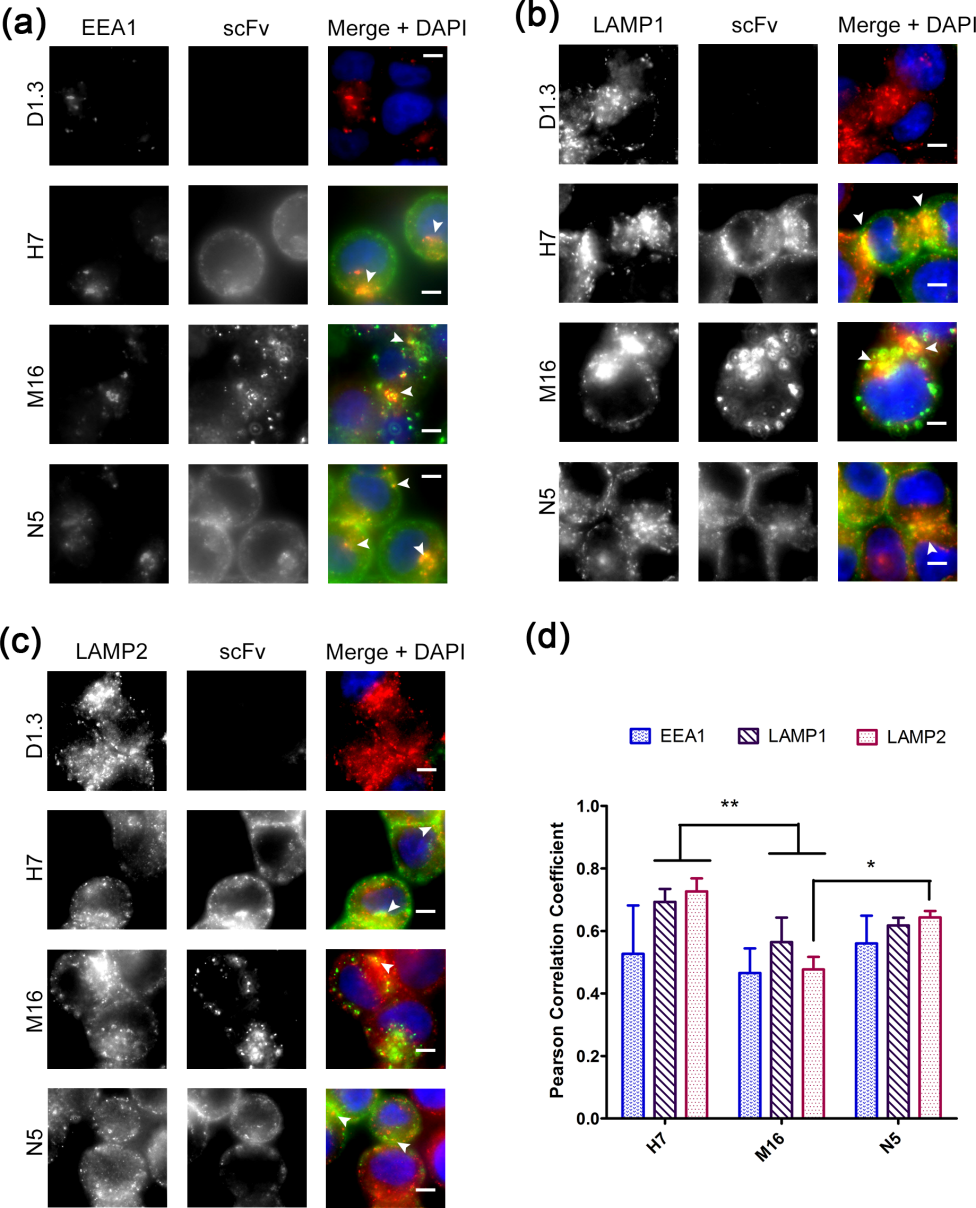
Intracellular co-localization of scFvs with endosomal and lysosomal markers. (a) SK-BR-3 cells which had been allowed to endocytose scFv dimers (green in merged) were counterstained with an antibody against early endosome antigen type 1 (EEA1, red in merged). (b and c) The same steps were used to counterstain with antibodies against lysosomal-associated membrane proteins 1 and 2 (LAMP1 and LAMP2, red in merged). As indicated by arrowheads, all scFvs co-localized with EEA1, LAMP1 and LAMP2. Scale bar is 5μm. (d) Co-localization with EEA1, LAMP1, and LAMP2 was quantified by Pearson correlation coefficient (**, p <0.01, *, p <0.05).

Finally, the co-localization of internalized scFvs with markers of intracellular compartments was evaluated to determine what alterations in antibody trafficking were responsible for the increased internalization behavior of M16. H7, M16 and N5, all showed isolated areas of co-localization with the endosomal marker EEA1 (Fig 6A), and the late-endosomal / lysosomal markers LAMP1 (Fig 6B) and LAMP2 (Fig 6C). Quantitative co-localization analysis did not suggest that pH-sensitivity dramatically shifted trafficking away from the lysosome (LAMP1 or LAMP2) and towards the endosome (EEA1), or vice versa (Fig 6D). Instead, the Pearson correlation coefficients suggested that co-localization of pH-sensitive M16 with each of the three intracellular markers was uniformly reduced compared with either H7 or N5, although only differences in LAMP1 and LAMP2 coefficients were statistically significant (Fig 6D, p <0.01). Reflective of the uniform reduction in co-localization with endosomal and lysosomal markers, the discrete foci of accumulated M16 within the cells did not visually appear to localize strongly with any of the markers tested (Fig 6A–6C).

## Discussion

Antibodies with the ability to respond to endosomal pH are intriguing because they offer an additional, tunable, layer of functionality beyond antigen binding affinity. Through semi-rational histidine saturation mutagenesis of parental anti-TfR scFv H7, combined with rapid screening via yeast surface display, it was possible to engineer appreciable increases in dissociation from TfR at pH 5.5, while largely maintaining pH 7.4 antigen binding. In particular, mutant M16 displayed increased overall cellular association primarily resulting from increased intracellular accumulation, and a substantially different intracellular distribution compared to wild-type H7 or the pH-insensitive mutant N5.

Here, we embraced the idea that pH-sensitivity results from multiple mutations acting in concert [43], especially from multiple histidines in close proximity [39,44,45,46]. After screening the histidine-saturated CDRH1 library, we found that the resulting pH-sensitive scFvs contained three or more histidines (out of 10 total residues), centrally located in CDRH1, supporting the beneficial effect of multiple proximal histidines for pH-responsiveness (Table 1). The protonation of the histidine residues at pH 5.5 drives an increased dissociation, also indicating that the CDRH1 loop likely participates in antigen binding as predicted prior to library design [33]. In addition, to achieve the multiplicity of histidine mutations that were key to the outcomes presented above, histidine saturation mutagenesis provides the most efficient approach. Our results were mirrored by an engineered version of the therapeutic IgG adalimumab, where the synergistic effect of paired histidine mutations in CDRs imparted pH-sensitive antigen binding [47]. The context of histidines within the antigen binding site is also an important factor as evidenced by an anti-HER2 Fc-Ab where pH-sensitivity resulted from non-histidine mutations proximal to wild-type histidines [48]. Given the prevalence of three or more histidine mutations in pH-responsive clones, the context-driven effects of such bystander mutations likely played less of a role in our study.

After binding at pH 7.4, scFv M16 accumulates at significantly higher levels than H7 or its pH-insensitive counterpart N5. As described above, models of anti-TfR immunotoxin delivery have pointed to TfR dissociation as an influential parameter in predicting increased cell-association [24,25]. The data presented here indicate that pH-dependent TfR dissociation properties of M16 lead to its distinct phenotypic properties. Since assays were performed at scFv concentrations capable of saturating cell surface TfR, the observed differences in internalization phenotype stemmed from differences in intracellular interactions. The unique property of M16 capable of reshaping intracellular interactions was its capacity for pH-dependent dissociation from TfR post-internalization. Inhibition of acidification of the endosomal pathway had the effect of completely eliminating enhanced M16 internalization and normalizing the internalization behavior to that of wild-type H7 (Fig 5C). Other studies have also indicated that dissociation of an antibody-TfR complex at endosomal pH can be an important property in governing TfR-mediated trafficking. When a number of parental anti-TfR antibodies having varying levels of intrinsic, pH-sensitive TfR binding were characterized, antibodies having reduced affinity for TfR at pH 5.5 also had substantially higher extents of transcytosis across an in vitro blood-brain barrier model [49]. In addition to pH-sensitivity, scFv M16 affinity at pH 7.4 was slightly attenuated (2.5-fold reduction) compared to H7 or N5, an output previously observed in the engineering of pH-sensitive binders [48]. While pH-sensitivity is playing the major role in differential trafficking of M16, we cannot completely rule out a minor contribution of M16’s lowered affinity at neutral pH. Recent studies have indicated that it was necessary to engineer a ~30-fold reduction of binding affinity at neutral pH (to ~600 nM) to alter trafficking and decrease lysosomal sorting of anti-TfR antibodies [50,51]. By contrast, the pH 7.4 affinity for M16 remains in the nanomolar range (Fig 3C), and monomeric and avidity-enhanced dimeric forms of M16 yielded the same distinct intracellular distribution (Fig 4B). In addition, it was previously shown that when anti-TfR antibodies having a range of affinity at neutral pH were tested, only those also endowed with pH-sensitive binding attributes were capable of differential trafficking and transport [49]. Finally, if it is desired to restore high affinity cell surface targeting in concert with efficient intracellular release, one could combine the pH-sensitive CDRH1 mutations present in M16 with mutations outside of CDRH1 that we previously demonstrated could improve the equilibrium binding affinity at pH 7.4 [33].

Endocytosed M16, H7, and N5 all co-localized with EEA1, LAMP1, and LAMP2 (Fig 6A–6C), frequently cited markers of early-endosomes and late-endosomes/lysosomes, respectively. Quantitative image analysis did not indicate an obvious shift in endosomal versus lysosomal trafficking for M16 (Fig 6D) compared to H7 or N5. However, an overall reduction in co-localization for all markers was observed. The reduction in M16 co-localization with LAMP1 and LAMP2 correlates with previous findings where reduced anti-TfR affinity, anti-TfR avidity or pH-sensitive TfR binding led to a reduction in lysosomal association [49,52,53]. Also, the reduction in M16 association with endosomal and lysosomal markers occurred in concert with the appearance of large vesicular structures that did not co-localize with any of the markers tested, suggesting a divergence in trafficking. Of interest, when previous studies compared a pH-insensitive anti-TfR antibody (128.1) to a pH-sensitive antibody (MEM-189), 128.1 was found to co-localize with CD63 (late endosomal/lysosomal maker) while MEM-189 instead accumulated in large intracellular structures, reminiscent of those observed with M16, that did not co-localize with CD63 [49]. In the future, more comprehensive trafficking analysis will be needed to definitively pinpoint the intracellular location of scFv M16. In summary, we have created a single chain-antibody, M16, which binds TfR with high affinity at pH7.4, and dissociates from TfR at pH 5.5 thereby markedly increasing intracellular accumulation, and potential therapeutic relevance. It is also anticipated that these antibodies will prove useful in understanding pH-dependent mechanisms underlying TfR-targeted antibody endocytosis and trafficking.

## Supporting Information

S1 Fig Flow cytometric screening of the CDRH1his library for scFvs lacking pH 5.5 sensitivity. In the first two panels (a and b), dot plots of H7 +TfR and pool F -TfR are shown after pH 5.5 antigen dissociation treatment. (c) CDRH1his library after pH 5.5 dissociation. The binding signal of H7, Pool F and CDRH1his at pH 5.5 provided reference points for sorting of the 5P, N and M pools. Gates are drawn for illustrative purposes. Pool 5P was derived from four rounds of sorting CDRH1his, selecting for scFvs that maintained binding at pH 5.5.
(TIF)

## References

[1] Edgcomb SP, Murphy KP (2002) Variability in the pKa of histidine side-chains correlates with burial within proteins. Proteins 49: 1–6. 12211010 10.1002/prot.10177

[2] Chan P, Warwicker J (2009) Evidence for the adaptation of protein pH-dependence to subcellular pH. BMC Biol 7: 69 10.1186/1741-7007-7-69 19849832 PMC2770037

[3] Roopenian DC, Akilesh S (2007) FcRn: the neonatal Fc receptor comes of age. Nat Rev Immunol 7: 715–725. 17703228 10.1038/nri2155

[4] Dall’Acqua WF, Woods RM, Ward ES, Palaszynski SR, Patel NK, Brewah YA, et al (2002) Increasing the Affinity of a Human IgG1 for the Neonatal Fc Receptor: Biological Consequences. J Immunol 169: 5171–5180. 12391234 10.4049/jimmunol.169.9.5171

[5] Dall'Acqua WF, Kiener PA, Wu H (2006) Properties of Human IgG1s Engineered for Enhanced Binding to the Neonatal Fc Receptor (FcRn). J Biol Chem 281: 23514–23524. 16793771 10.1074/jbc.M604292200

[6] Oganesyan V, Damschroder MM, Woods RM, Cook KE, Wu H, Dall’Acqua WF (2009) Structural characterization of a human Fc fragment engineered for extended serum half-life. Mol Immunol 46: 1750–1755. 10.1016/j.molimm.2009.01.026 19250681

[7] Vaccaro C, Zhou J, Ober RJ, Ward ES (2005) Engineering the Fc region of immunoglobulin G to modulate in vivo antibody levels. Nat Biotech 23: 1283–1288. 10.1038/nbt1143 16186811

[8] Igawa T, Ishii S, Tachibana T, Maeda A, Higuchi Y, Shimaoka S, et al (2010) Antibody recycling by engineered pH-dependent antigen binding improves the duration of antigen neutralization. Nat Biotech 28: 1203–1207. 10.1038/nbt.1691 20953198

[9] Chaparro-Riggers J, Liang H, DeVay RM, Bai L, Sutton JE, Chen W, et al (2012) Increasing Serum Half-life and Extending Cholesterol Lowering in Vivo by Engineering Antibody with pH-sensitive Binding to PCSK9. J Biol Chem 287: 11090–11097. 10.1074/jbc.M111.319764 22294692 PMC3322827

[10] Sarkar CA, Lowenhaupt K, Horan T, Boone TC, Tidor B, Lauffenburger DA (2002) Rational cytokine design for increased lifetime and enhanced potency using pH-activated [ldquo]histidine switching[rdquo]. Nat Biotech 20: 908–913. 10.1038/nbt725 12161759

[11] Yoon DJ, Chu DSH, Ng CW, Pham EA, Mason AB, Hudson DM, et al (2009) Genetically engineering transferrin to improve its in vitro ability to deliver cytotoxins. J Control Release 133: 178–184. 10.1016/j.jconrel.2008.10.006 18992290 PMC2681226

[12] Daniels TR, Delgado T, Rodriguez JA, Helguera G, Penichet ML (2006) The transferrin receptor part I: Biology and targeting with cytotoxic antibodies for the treatment of cancer. Clin Immunol 121: 144–158. 16904380 10.1016/j.clim.2006.06.010

[13] Daniels TR, Delgado T, Helguera G, Penichet ML (2006) The transferrin receptor part II: Targeted delivery of therapeutic agents into cancer cells. Clin Immunol 121: 159–176. 16920030 10.1016/j.clim.2006.06.006

[14] Sun H, Cox MC, Li H, Mason AB, Woodworth RC, Sadler PJ (1998) [1H,13C] NMR determination of the order of lobe loading of human transferrin with iron: comparison with other metal ions. FEBS Lett 422: 315–320. 9498807 10.1016/s0014-5793(98)00034-9

[15] Wally J, Halbrooks PJ, Vonrhein C, Rould MA, Everse SJ, Mason AB, et al (2006) The Crystal Structure of Iron-free Human Serum Transferrin Provides Insight into Inter-lobe Communication and Receptor Binding. J Biol Chem 281: 24934–24944. 16793765 10.1074/jbc.M604592200 PMC1895924

[16] Mayle KM, Le AM, Kamei DT (2012) The intracellular trafficking pathway of transferrin. Biochimica et Biophysica Acta (BBA)—General Subjects 1820: 264–281. 21968002 10.1016/j.bbagen.2011.09.009 PMC3288267

[17] Leverence R, Mason AB, Kaltashov IA (2010) Noncanonical interactions between serum transferrin and transferrin receptor evaluated with electrospray ionization mass spectrometry. Proc Natl Acad Sci U S A 107: 8123–8128. 10.1073/pnas.0914898107 20404192 PMC2889525

[18] Gumerov DR, Mason AB, Kaltashov IA (2003) Interlobe Communication in Human Serum Transferrin: Metal Binding and Conformational Dynamics Investigated by Electrospray Ionization Mass Spectrometry. Biochemistry 42: 5421–5428. 12731884 10.1021/bi020660b

[19] Eckenroth BE, Steere AN, Chasteen ND, Everse SJ, Mason AB (2011) How the binding of human transferrin primes the transferrin receptor potentiating iron release at endosomal pH. Proc Natl Acad Sci U S A 108: 13089–13094. 10.1073/pnas.1105786108 21788477 PMC3156180

[20] Ciechanover A, Schwartz AL, Dautry-Varsat A, Lodish HF (1983) Kinetics of internalization and recycling of transferrin and the transferrin receptor in a human hepatoma cell line. Effect of lysosomotropic agents. J Biol Chem 258: 9681–9689. 6309781

[21] Luck AN, Mason AB (2013) Structure and dynamics of drug carriers and their interaction with cellular receptors: Focus on serum transferrin. Adv Drug Del Rev 65: 1012–1019. 10.1016/j.addr.2012.11.001 PMC3602139 23183585

[22] Lao BJ, Tsai W-LP, Mashayekhi F, Pham EA, Mason AB, Kamei DT (2007) Inhibition of transferrin iron release increases in vitro drug carrier efficacy. J Control Release 117: 403–412. 17239470 10.1016/j.jconrel.2006.12.001 PMC2034207

[23] Wenning LA, Yazdi PT, Murphy RM (1998) Quantitative analysis of protein synthesis inhibition and recovery in CRM107 immunotoxin-treated HeLa cells. Biotechnol Bioeng 57: 484–496. 10099226 10.1002/(sici)1097-0290(19980220)57:4<484::aid-bit13>3.0.co;2-c

[24] Yazdi PT, Murphy RM (1994) Quantitative Analysis of Protein Synthesis Inhibition by Transferrin-Toxin Conjugates. Cancer Res 54: 6387–6394. 7987833

[25] Yazdi PT, Wenning LA, Murphy RM (1995) Influence of Cellular Trafficking on Protein Synthesis Inhibition of Immunotoxins Directed against the Transferrin Receptor. Cancer Res 55: 3763–3771. 7641191

[26] Boder ET, Wittrup KD (1997) Yeast surface display for screening combinatorial polypeptide libraries. Nat Biotechnol 15: 553–557. 9181578 10.1038/nbt0697-553

[27] Wentz AE, Shusta EV (2007) A Novel High-Throughput Screen Reveals Yeast Genes That Increase Secretion of Heterologous Proteins. Appl Environ Microbiol 73: 1189–1198. 17189442 10.1128/AEM.02427-06 PMC1828678

[28] Shusta EV, Raines RT, Pluckthun A, Wittrup KD (1998) Increasing the secretory capacity of Saccharomyces cerevisiae for production of single-chain antibody fragments. Nat Biotech 16: 773–777. 10.1038/nbt0898-773 9702778

[29] Piatesi A, Howland SW, Rakestraw JA, Renner C, Robson N, Cebon J, et al (2006) Directed evolution for improved secretion of cancer–testis antigen NY-ESO-1 from yeast. Protein Expr Purif 48: 232–242. 16563796 10.1016/j.pep.2006.01.026

[30] Hackel BJ, Huang D, Bubolz JC, Wang XX, Shusta EV (2006) Production of soluble and active transferrin receptor-targeting single-chain antibody using Saccharomyces cerevisiae. Pharm Res 23: 790–797. 16550469 10.1007/s11095-006-9778-7

[31] Poul M-A, Becerril B, Nielsen UB, Morisson P, Marks JD (2000) Selection of tumor-specific internalizing human antibodies from phage libraries. J Mol Biol 301: 1149–1161. 10966812 10.1006/jmbi.2000.4026

[32] Van Antwerp JJ, Wittrup KD (2000) Fine Affinity Discrimination by Yeast Surface Display and Flow Cytometry. Biotechnol Prog 16: 31–37. 10662486 10.1021/bp990133s

[33] Tillotson BJ, de Larrinoa IF, Skinner CA, Klavas DM, Shusta EV (2013) Antibody affinity maturation using yeast display with detergent-solubilized membrane proteins as antigen sources. Protein Eng Des Sel 26: 101–112. 10.1093/protein/gzs077 23109730 PMC3542525

[34] Benatuil L, Perez JM, Belk J, Hsieh C-M (2010) An improved yeast transformation method for the generation of very large human antibody libraries. Protein Eng Des Sel 23: 155–159. 10.1093/protein/gzq002 20130105

[35] Swers JS, Kellogg BA, Wittrup KD (2004) Shuffled antibody libraries created by in vivo homologous recombination and yeast surface display. Nucleic Acids Res 32: e36–e36. 14978223 10.1093/nar/gnh030 PMC373425

[36] Gietz RD, Schiestl RH (2007) High-efficiency yeast transformation using the LiAc/SS carrier DNA/PEG method. Nat Protocols 2: 31–34. 17401334 10.1038/nprot.2007.13

[37] Wang XX, Cho YK, Shusta EV (2007) Mining a yeast library for brain endothelial cell-binding antibodies. Nat Methods 4: 143–145. 17206151 10.1038/nmeth993 PMC2637222

[38] Dautry-Varsat A, Aaron C, Lodish HF (1983) pH and the Recycling of Transferrin during Receptor-Mediated Endocytosis. Proc Natl Acad Sci U S A 80: 2258–2262. 6300903 10.1073/pnas.80.8.2258 PMC393798

[39] Murtaugh ML, Fanning SW, Sharma TM, Terry AM, Horn JR (2011) A combinatorial histidine scanning library approach to engineer highly pH-dependent protein switches. Protein Sci 20: 1619–1631. 10.1002/pro.696 21766385 PMC3190156

[40] Chitambar CR, Zivkovic Z (1989) Release of soluble transferrin receptor from the surface of human leukemic HL60 cells. Blood 74: 602–608. 2752136

[41] Bowman EJ, Siebers A, Altendorf K (1988) Bafilomycins: a class of inhibitors of membrane ATPases from microorganisms, animal cells, and plant cells. Proc Natl Acad Sci U S A 85: 7972–7976. 2973058 10.1073/pnas.85.21.7972 PMC282335

[42] Johnson LS, Dunn KW, Pytowski B, McGraw TE (1993) Endosome acidification and receptor trafficking: bafilomycin A1 slows receptor externalization by a mechanism involving the receptor's internalization motif. Mol Biol Cell 4: 1251–1266. 8167408 10.1091/mbc.4.12.1251 PMC275762

[43] Gera N, Hill AB, White DP, Carbonell RG, Rao BM (2012) Design of pH sensitive binding proteins from the hyperthermophilic Sso7d scaffold. PLoS One 7: e48928 10.1371/journal.pone.0048928 23145025 PMC3492137

[44] Kulkarni MV, Tettamanzi MC, Murphy JW, Keeler C, Myszka DG, Chayen NE, et al (2010) Two Independent Histidines, One in Human Prolactin and One in Its Receptor, Are Critical for pH-dependent Receptor Recognition and Activation. J Biol Chem 285: 38524–38533. 10.1074/jbc.M110.172072 20889499 PMC2992285

[45] Martin WL, West AP Jr, Gan L, Bjorkman PJ (2001) Crystal Structure at 2.8 Å of an FcRn/Heterodimeric Fc Complex: Mechanism of pH-Dependent Binding. Mol Cell 7: 867–877. 11336709 10.1016/s1097-2765(01)00230-1

[46] Giannetti AM, Halbrooks PJ, Mason AB, Vogt TM, Enns CA, Bjorkman PJ (2005) The molecular mechanism for receptor-stimulated iron release from the plasma iron transport protein transferrin. Structure 13: 1613–1623. 16271884 10.1016/j.str.2005.07.016

[47] Schröter C, Günther R, Rhiel L, Becker S, Toleikis L, Doerner A, et al (2014) A generic approach to engineer antibody pH-switches using combinatorial histidine scanning libraries and yeast display. mAbs 7: 138–151. 10.4161/19420862.2014.985993 PMC4622719 25523975

[48] Traxlmayr MW, Lobner E, Hasenhindl C, Stadlmayr G, Oostenbrink C, Rüker F, et al (2014) Construction of pH-sensitive Her2-binding IgG1-Fc by directed evolution. Biotechnology Journal 9: 1013–1022. 10.1002/biot.201300483 24964247 PMC4314675

[49] Sade H, Baumgartner C, Hugenmatter A, Moessner E, Freskgård P-O, Niewoehner J (2014) A Human Blood-Brain Barrier Transcytosis Assay Reveals Antibody Transcytosis Influenced by pH-Dependent Receptor Binding. PLoS One 9: e96340 10.1371/journal.pone.0096340 24788759 PMC4005765

[50] Atwal JK, Chen Y, Chiu C, Mortensen DL, Meilandt WJ, Liu Y, et al (2011) A Therapeutic Antibody Targeting BACE1 Inhibits Amyloid-β Production in Vivo. Sci Transl Med 3: 84ra43–84ra43. 10.1126/scitranslmed.3002254 21613622

[51] Yu YJ, Zhang Y, Kenrick M, Hoyte K, Luk W, Lu Y, et al (2011) Boosting Brain Uptake of a Therapeutic Antibody by Reducing Its Affinity for a Transcytosis Target. Sci Transl Med 3: 84ra44–84ra44. 10.1126/scitranslmed.3002230 21613623

[52] Bien-Ly N, Yu YJ, Bumbaca D, Elstrott J, Boswell CA, Zhang Y, et al (2014) Transferrin receptor (TfR) trafficking determines brain uptake of TfR antibody affinity variants. J Exp Med 211: 233–244. 10.1084/jem.20131660 24470444 PMC3920563

[53] Niewoehner J, Bohrmann B, Collin L, Urich E, Sade H, Maier P, et al (2014) Increased Brain Penetration and Potency of a Therapeutic Antibody Using a Monovalent Molecular Shuttle. Neuron 81: 49–60. 10.1016/j.neuron.2013.10.061 24411731

