# Supplementary figures and images for: Engineering an Anti-Transferrin Receptor ScFv for pH-Sensitive Binding Leads to Increased Intracellular Accumulation
Source: PLoS One. 2015 Dec 29;10(12):e0145820. doi: 10.1371/journal.pone.0145820 (PMC4694649; doi:10.1371/journal.pone.0145820)

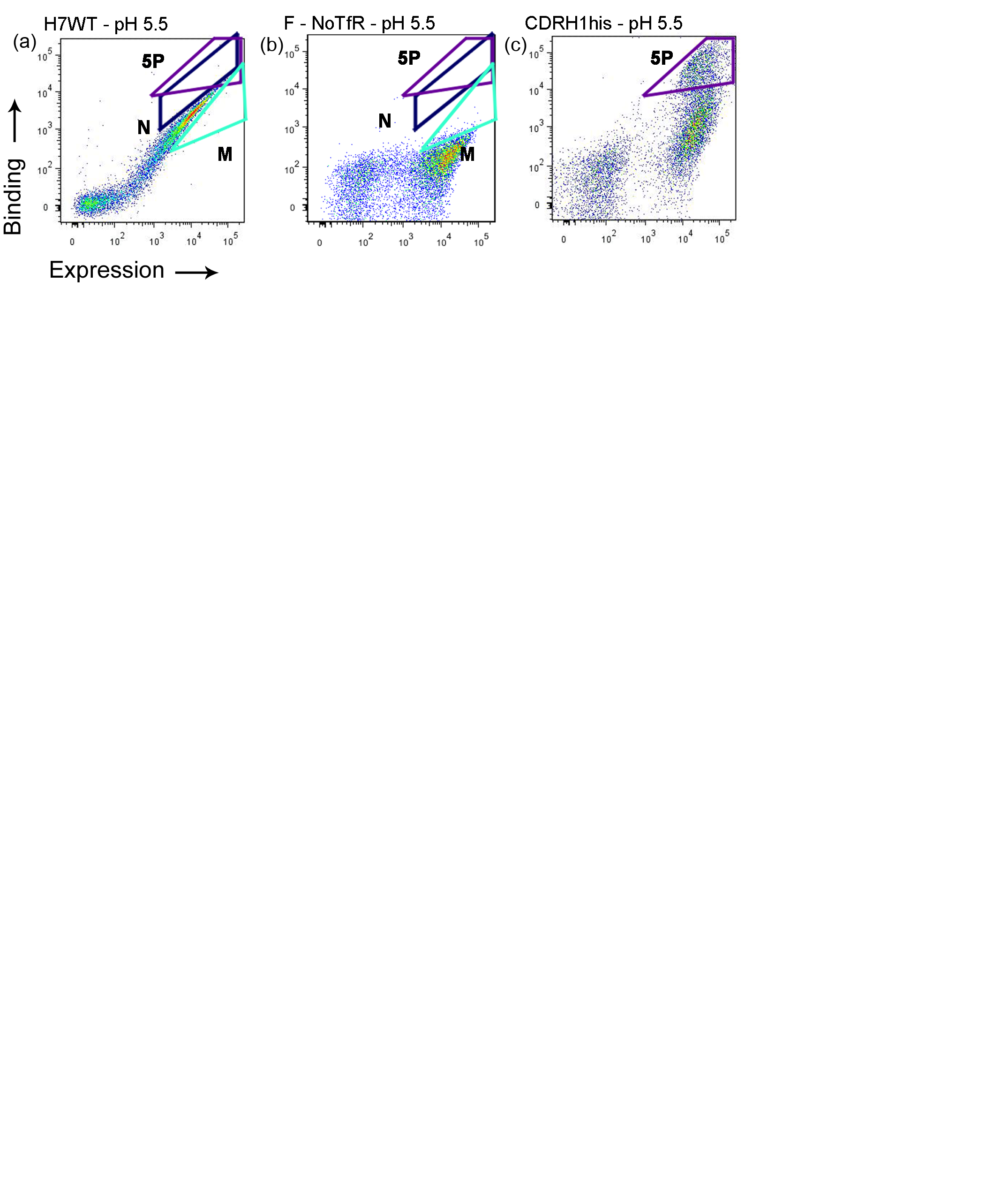

Supplement: S1 Fig — In the first two panels (a and b), dot plots of H7 +TfR and pool F -TfR are shown after pH 5.5 antigen dissociation treatment. (c) CDRH1his library after pH 5.5 dissociation. The binding signal of H7, Pool F and CDRH1his at pH 5.5 provided reference points for sorting of the 5P, N and M pools. Gates are drawn for illustrative purposes. Pool 5P was derived from four rounds of sorting CDRH1his, selecting for scFvs that maintained binding at pH 5.5. (TIF) [file pone.0145820.s001.tif]
